# EED-CL: Extended EEG Deformer with Contrastive Learning for Robust Emotion Recognition

**DOI:** 10.3390/bioengineering13010029

**Published:** 2025-12-26

**Authors:** Hyoung-Gook Kim, Jin-Young Kim

**Affiliations:** 1 Department of Electronic Convergence Engineering, Kwangwoon University, 20 Gwangun-ro, Nowon-gu, Seoul 01897, Republic of Korea; 2 Department of Intelligent Electronics and Computer Engineering, Chonnam National University, 77 Yongbong-ro, Buk-gu, Gwangju 61186, Republic of Korea; beyondi@jnu.ac.kr

**Keywords:** emotion recognition, electroencephalography, EEG-Deformer, contrastive learning

## Abstract

Emotion recognition based on EEG signals remains a challenging task due to the complex spatiotemporal properties of brain activity and substantial intersubject variability. To address these challenges, we propose the EED-CL framework, which integrates an extended EEG-Deformer (EED) with contrastive learning (CL). The proposed model incorporates a depthwise separable convolution encoder for efficient extraction of spatiotemporal EEG features, a hierarchical coarse-to-medium-to-fine (HCMFT) transformer to capture multiscale temporal patterns, and an attentive dense information purification (ADIP) module to suppress noise and refine essential latent representations. In addition, CL-based pretraining facilitates robust feature learning even in settings with limited labeled data. The extracted multiscale features are integrated and classified through a Transformer encoder and an MLP. Experiments conducted on multiple benchmark EEG datasets show that EED consistently outperforms conventional models, while EED-CL achieves further improvements under label-constrained conditions. Notably, EED-CL demonstrates strong robustness to intersubject variability and noise, enabling stable emotion classification even when labeled samples are scarce. These findings indicate that EED-CL effectively captures multiscale spatiotemporal EEG patterns and offers a scalable and reliable approach for EEG-based emotion recognition.

## 1. Introduction

Electroencephalography (EEG) is a technology that noninvasively measures the electrical activity of the brain, enabling real-time inference of human emotional states. EEG-based emotion recognition has broad applications in mental health monitoring, human–computer interactions, education, and gaming. Compared to other physiological signals, EEG is relatively robust to external environmental factors and allows for a more precise classification of emotions by analyzing brain activity patterns that reflect psychological and physiological states of an individual [1,2].

However, in contrast to other biosignals, EEG signals have small amplitudes, and brain activity patterns vary significantly among subjects. EEG data exhibit multidimensional interactions across channels and are highly susceptible to external noise and motion artifacts. Moreover, emotion-related brain activity requires the simultaneous consideration of temporal and spatial correlations [3,4]. Because of these characteristics, traditional machine learning-based approaches struggle to capture the fine-grained temporal variations and spatial relationships inherent in EEG signals [5].

The introduction of deep learning has marked a crucial turning point for overcoming these limitations. Convolutional neural network (CNN)-based models have been widely employed for EEG emotion recognition because they can simultaneously learn spatial and temporal patterns [6,7]. However, CNNs possess a limited receptive field, rendering it difficult to capture long-term temporal dependencies or integrate temporal patterns across multiple scales [8,9]. Recurrent neural network (RNN)-based architectures, including long short-term memory (LSTM) and gated recurrent units (GRUs) [10,11], are effective for learning long-term dependencies, but have difficulty in modeling spatial interactions across EEG channels. In addition, they offer limited parallelization, and as the sequence length increases, both the computational complexity and memory requirements increase substantially.

To address these limitations, transformer-based approaches capable of simultaneously capturing long-term dependencies and global correlations have gained significant attention for EEG-based emotion recognition [12,13]. The EEG-Conformer model [14], which integrates a transformer module, is designed to learn both local and global features concurrently, allowing the joint processing of short- and long-term temporal patterns. Nevertheless, it makes limited use of intermediate latent features and demonstrates restricted generalization performance in low-label environments [15].

To address these limitations, an EEG-Deformer [16] was proposed. The model employs a shallow CNN-based encoder in the initial stage and introduces a hierarchical coarse-to-fine transformer (HCFT) structure to capture coarse and fine temporal patterns jointly. In addition, a dense information purification (DIP) module refines and integrates the intermediate representations, achieving improved emotion recognition performance compared with conventional CNN/transformer-based models. However, the architecture still faces the following limitations: intermediate representations may not contribute sufficiently to the final classification; noise discrimination capability is constrained; and some critical information may be lost. Moreover, it has low parameter efficiency and high computational cost.

Another major challenge in EEG-based emotion recognition is limited generalization under low-label settings. EEG data exhibit high intersubject variability, which makes it challenging to obtain sufficient training data, potentially causing models to be biased toward specific subjects or experimental conditions. Recent studies have highlighted contrastive learning (CL) [17] as a promising approach to address this limitation. CL enables the learning of similarities and differences between signals, even in unlabeled data, allowing the acquisition of feature-invariant representations that are robust to intersubject variability and noise [18]. This approach demonstrates the potential for stable emotion classification in low-label environments, while enabling more robust learning of latent EEG patterns.

To overcome the limitations of existing EEG-based models and improve emotion recognition, we propose EED-CL, which combines an extended EEG-Deformer (EED) with contrastive learning (CL). The proposed model consists of several key components, each addressing specific challenges in EEG representation learning.

First, a depthwise separable convolution-based shallow feature encoder (DSC-SFE) [19] was employed to effectively disentangle and learn the temporal and spatial patterns of EEG signals. This design reduces the number of parameters, enabling computationally efficient and stable feature extraction, particularly in data-limited environments.

Second, the original HCFT is extended to a hierarchical coarse–medium–fine transformer (HCMFT). EEG signals are divided into three levels—coarse, medium, and fine—allowing the model to learn and integrate long-term, intermediate, and fine-grained temporal patterns. This hierarchical design effectively captures multiscale temporal information, which is critical for accurate emotion recognition.

Third, the existing DIP module is enhanced into an attentive dense information purification (ADIP) module. ADIP emphasizes essential information while suppressing irrelevant noise at medium and fine scales. This maximizes the utilization of intermediate latent features and enhances the discriminative power of the final EEG representation.

In addition, CL-based pretraining is applied to strengthen the consistency of EEG feature representations under limited-label conditions. This approach increases robustness to intersubject variability, enabling stable emotion classification even with scarce data.

Finally, multiscale features extracted from the HCMFT and ADIP modules are integrated, and the final emotion labels are predicted using a Transformer encoder followed by an MLP classifier. Together, these components ensure that EED-CL captures complex temporal-spatial EEG patterns, effectively reduces noise, and achieves high recognition accuracy with robust generalization.

The remainder of this paper is organized as follows: In Section 2, related works are reviewed. In Section 3, the proposed method is described in detail. Section 4 presents the experimental results, and Section 5 discusses the findings and limitations of the study. Finally, Section 6 concludes the paper and suggests possible directions for future research.

## 2. Related Works

Research on EEG-based emotion recognition has evolved from traditional machine-learning approaches to deep learning, hybrid, and self-supervised learning frameworks. This section reviews these approaches by focusing on their methodological characteristics, major achievements, and inherent limitations.

**Traditional feature-based approaches**: Early studies manually extracted features from EEG signals, such as the power spectral density in the frequency domain, wavelet coefficients, and various statistical features. Classical classifiers, such as support vector machines (SVMs), k-nearest neighbors, and decision trees, have been employed for emotion classification. These methods are advantageous in terms of interpretability and ease of implementation. However, they failed to fully capture the complex time–frequency correlations and nonlinear characteristics of EEG data and were highly sensitive to noise and intersubject variability [2,20].

**CNN-based deep learning approaches**: The emergence of deep learning has provided a breakthrough in overcoming the limitations of traditional methods. CNN-based models can automatically learn spatial and temporal patterns from EEG signals and have demonstrated stable performance, even in limited-data scenarios. A representative model, EEGNet, employs depthwise separable convolution to improve parameter efficiency and robustly extract spatiotemporal features [21]. However, CNN-only models have a limited ability to learn long-term temporal dependencies because of their fixed receptive field, and they struggle to integrate temporal information across multiple scales. To address these issues, MSDCGTNet combines multiscale dynamic 1D CNNs with a gated transformer to jointly capture spatial–spectral representations and global temporal patterns [22].

**RNN-based approaches**: RNN-based models have been widely employed in EEG-based emotion recognition owing to their ability to capture long-term temporal dependencies. Variants based on LSTM and GRU, particularly bidirectional LSTM (BLSTM) [23] and bidirectional GRU (BGRU) [24], have been shown to enhance the performance by learning temporal information in both directions. To address the difficulties associated with long sequences, several studies have proposed CNN-RNN hybrid architectures that simultaneously learn spatial and temporal features. These approaches effectively mitigate the structural limitations of RNNs, integrate short- and long-term patterns, and improve the accuracy and generalization of EEG-based emotion recognition.

**Transformer and hybrid models**: Transformer-based and hybrid architectures have recently emerged as key trends in EEG emotion recognition. The EEG-Conformer integrates the CNN and transformer modules in a series to learn local and global features jointly. Guo et al. combined a Transformer encoder with depthwise convolution to further improve EEG emotion recognition performance [25]. Similarly, ERTNet proposed a CNN-transformer hybrid model designed to improve interpretability, whereas EmoSTT introduced a pure transformer architecture capable of emotion classification directly from raw EEG inputs without preprocessing [26]. In this context, the previously introduced EEG-Deformer integrates a CNN-based feature encoder with an HCFT to learn short- and long-term temporal patterns jointly.

**Self-supervised and CL approaches**: In actual EEG-based emotion recognition studies, various CL-based frameworks have been applied to EEG-based emotion recognition studies. For example, cross-subject CL minimizes intersubject EEG signal variability and improves emotion recognition accuracy [17], whereas dual-branch self-supervised CL simultaneously learns temporal and frequency-domain information to enhance performance [18]. In addition, self-supervised group meiosis CL and joint contrastive learning with feature alignment have been shown to effectively improve generalization performance in label-limited environments [27]. These studies demonstrate that CL-based approaches are effective in learning representations that are robust to intersubject variability and noise in EEG emotion recognition.

**Multimodal and graph-based approaches**: In recent years, multimodal learning, which combines EEG with other physiological or behavioral signals such as electrocardiograms (ECG), galvanic skin responses (GSR), or facial images, has attracted increasing attention [28]. Additionally, graph-based methods that model connectivity between EEG electrodes using graph neural networks have recently been proposed [29,30]. Models such as DAGAM and GMSS handle intersubject variability and integrate self-supervised or CL to enhance the generalization capability of learned representations [31,32].

Research on EEG-based emotion recognition has evolved from handcrafted feature extraction to CNNs, RNNs, Transformers, graph-based networks, self-supervised learning, and multimodal frameworks. Building upon these research trends, this study proposed the EED-CL approach, which aims to address the structural and learning limitations of the original EEG-Deformer and achieve robust emotion recognition performance even under limited-label conditions.

## 3. Proposed EEG-Based Emotion Recognition Methods

### 3.1. The Overview of the Proposed System

**Figure 1:**
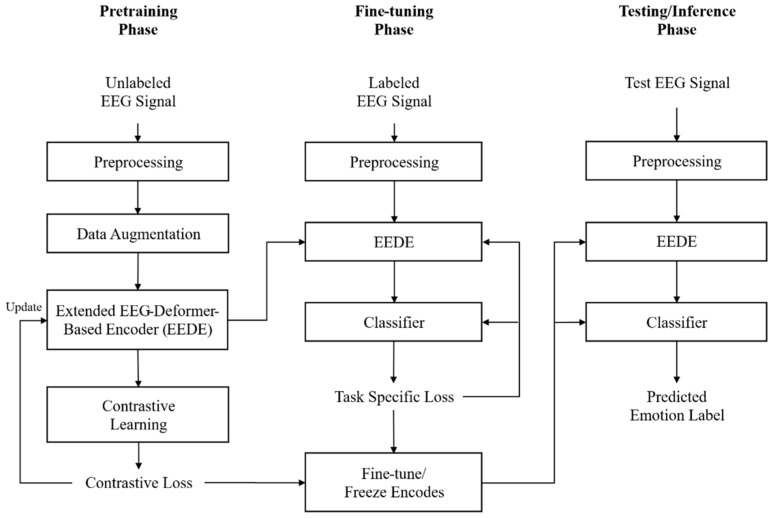
Overall architecture of the proposed system.

This section presents an overview of the training and inference pipeline of the proposed EEG-based emotion recognition system, which leverages CL and the EED. As illustrated in Figure 1, the system comprises three interconnected stages: pretraining, fine-tuning, and inference, covering the entire process from EEG data acquisition to real-time emotion recognition.

EEG signals are collected from two sources, including a large-scale offline EEG dataset and real-time online EEG streams. Both data types are processed using a unified preprocessing pipeline to ensure consistent model input. The preprocessing procedure includes a 4–47 Hz bandpass filter for noise suppression, interelectrode reference recalibration to reduce measurement bias, and automatic removal of physiological artifacts such as ocular and motion artifacts using a stacked sparse autoencoder [33]. The processed signals are then normalized to produce clean EEG time-series data suitable for downstream modeling.

In the pretraining phase, which forms the core of the proposed system, representation learning is conducted using CL. Various data augmentation techniques, including noise injection, temporal shifting, and filtering, are applied to preprocessed EEG signals to generate distinct variants from the same signal. Augmented EEG segments originating from the same emotion class are defined as positive pairs, whereas segments from different emotion classes are treated as negative pairs. The EED encoder is trained using the InfoNCE loss, which encourages positive pairs to be closer and negative pairs to be further apart in the embedding space, enabling the model to learn generalized spatiotemporal EEG representations. These learned representations are subsequently projected into a high-dimensional embedding space through a projection head.

During the fine-tuning stage, the pretrained encoder is adapted to labeled EEG emotion datasets to improve classification performance. To preserve the learned representations, a subset of the encoder parameters is frozen or updated using a reduced learning rate. A transformer-based MLP classifier is then trained using a weighted cross-entropy loss [34], which effectively alleviates class imbalance without relying on oversampling.

In the inference phase, the trained model performs emotion recognition in real-time or batch settings. EEG signals are segmented into 4 s sliding windows with a 50% overlap, and emotion predictions are generated for each window. The final emotion labels are determined by averaging the probabilities across the windows in a postprocessing step.

### 3.2. Extended EEG-Deformer-Based Encoder

**Figure 2:**
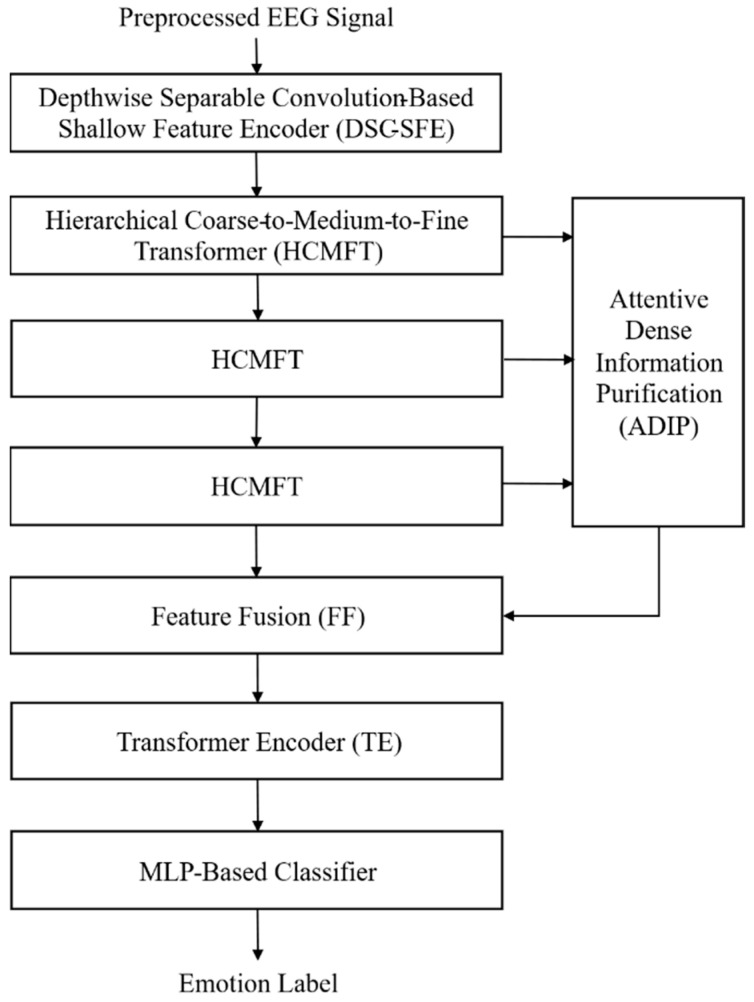
Network structure of the extended EEG-Deformer.

To address the structural limitations of conventional EEG-Deformer and achieve more precise and robust emotion recognition, this study proposes an extended EEG-Deformer. As illustrated in Figure 2, the proposed architecture comprises five key components: a depthwise separable convolution-based shallow feature encoder (DSC-SFE), an HCMFT module, an ADIP module, a Transformer encoder, and an MLP classifier.

Given the preprocessed EEG signals, the DSC-SFE module extracts low-level spatiotemporal features by leveraging depthwise and pointwise convolutions to capture fine-grained temporal dynamics and spatial correlations. This design reduces computational overhead and mitigates information loss, enabling stable high-level feature learning in subsequent transformer-based modules.

The extracted features are subsequently fed into the HCMFT module to learn hierarchical multiscale representations by partitioning the input into coarse, medium, and fine temporal levels.

The ADIP module then applies an attention mechanism to medium and fine-level features to dynamically assess their importance, enhancing emotion-relevant information while suppressing nonessential artifacts such as electromyography (EMG) and electrooculography (EOG), ultimately improving emotion classification performance.

Feature maps from the HCMFT and ADIP modules are fused to exploit hierarchical complementarity, after which a Transformer encoder refines the combined representation by modeling deep spatiotemporal dependencies and feature interactions. The refined features are ultimately classified by an MLP to generate emotion predictions.

#### 3.2.1. Depthwise Separable Convolution-Based Shallow Feature Encoder (DSC-SFE)

Conventional SFE relies on 2D convolution and max pooling, resulting in high computational complexity, a large number of parameters, and limitations such as information loss and incomplete separation of spatiotemporal features. These structural constraints hinder the accurate modeling of small temporal amplitude variations and interchannel spatial interactions in EEG signals.

**Figure 3:**
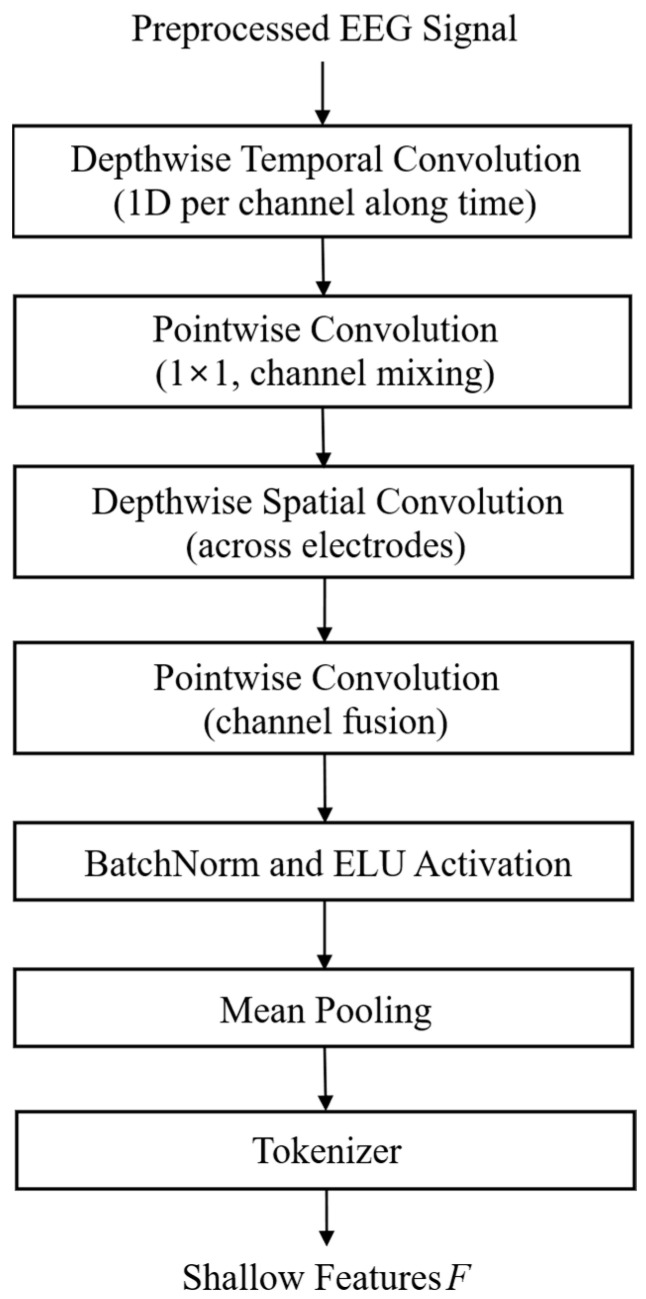
Structure of the shallow feature encoder.

To address these issues, we proposed a DSC-SFE that decomposes 2D convolutions into depthwise and pointwise (1 × 1) convolutions, maximizing computational efficiency while distinctly capturing temporal and spatial features. Figure 3 illustrates the structure of DSC-SFE, which receives EEG signals composed of *C* channels and *T* time samples as inputs. The module first applies depthwise temporal convolution, where independent 1D filters per channel precisely capture the temporal variation patterns. Owing to the lack of filter sharing across channels, the unique signal characteristics of each electrode are preserved, allowing the sensitive detection of small amplitude changes associated with emotional states. Pointwise convolution is then applied to integrate temporally extracted features across channels, enabling the learning of spatial interactions and synchronization patterns among the electrodes. Subsequently, depthwise spatial convolution captures interelectrode spatial correlations, followed by another pointwise convolution that consolidates temporal and spatial filter outputs to generate rich and comprehensive spatiotemporal representations.

After each convolutional operation, batch normalization is applied to mitigate training instability arising from interchannel statistical variations, and the exponential linear unit (ELU) activation function [35] is used to preserve both positive and negative EEG amplitudes. Subsequently, mean pooling is performed to compress the temporal and spatial dimensions while retaining critical patterns, reducing information loss compared with max pooling, and effectively preserving small amplitude variations and emotional cues. Finally, a learnable patch-embedding-based tokenizer converts the refined feature maps into token sequences suitable for transformer input, maintaining temporal order and sequential consistency across the EEG signal.

Overall, DSC-SFE significantly reduces the computational complexity and number of parameters compared to conventional SFE, while capturing temporal and spatial features with high fidelity. These properties mitigate overfitting, enhance interpretability, and provide a stable and efficient foundation for subsequent modules, including HCMFT and ADIP, thereby enabling effective learning of high-level emotional representations from EEG signals.

#### 3.2.2. Hierarchical Coarse-to-Medium-to-Fine Transformer (HCMFT)

**Figure 4:**
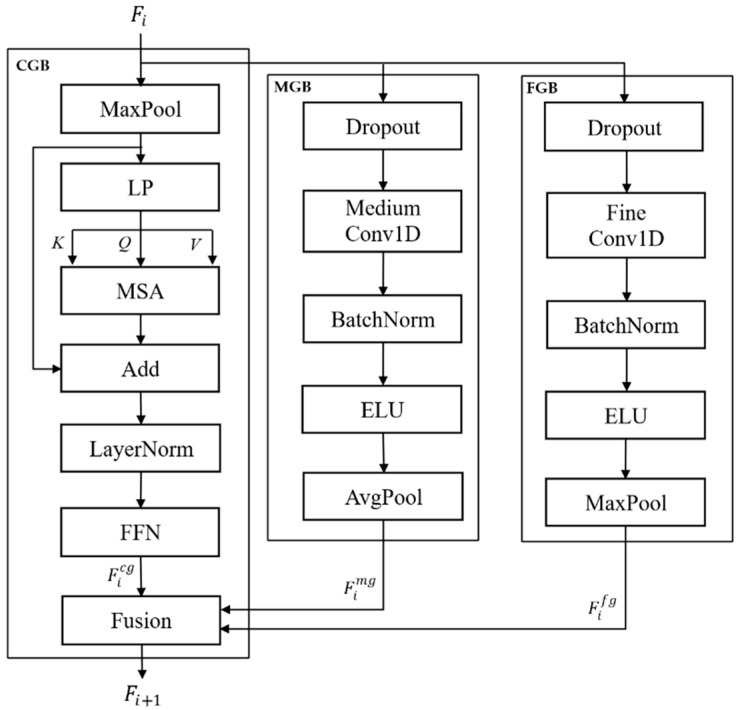
Structure of the hierarchical coarse-to-medium-to-fine transformer.

The conventional HCFT includes only the coarse-grained branch (CGB) and fine-grained branch (FGB), enabling the learning of long- and short-term patterns but failing to capture medium-scale temporal patterns. This limitation restricts the model to learn gradual emotional transitions and medium-duration event-related dynamics. To address this issue, this study proposes the HCMFT, which introduces an additional medium-grained branch (MGB) (as illustrated in Figure 4). Based on the tokenized EEG inputs, HCMFT hierarchically extracts features across three temporal resolutions: coarse, medium, and fine.

CGB employs a long temporal window and a low sampling rate to capture the long-term trends and global temporal dynamics of EEG signals. The MGB uses a medium-length window to model gradual emotional transitions and intermediate temporal interactions, whereas the FGB uses a short window and high-resolution signals to accurately capture transient variations and high-frequency characteristics. Operating in parallel, these three branches hierarchically learn features across multiple temporal scales and integrate them into a unified, fine-grained representation of the EEG temporal information.

In contrast, conventional CGB, based on multihead self-attention (MSA), effectively models global temporal dependencies but suffers from information dilution in critical regions and a high computational cost. To address these issues, HCMFT incorporates multiscale gated linear attention (MG-LA) [36], where each attention head is specialized for a distinct temporal scale, and a learnable gate, $G_{s}$, selectively emphasizes informative temporal segments. This design enables the efficient extraction of both long-term dependencies and salient emotional cues. The MG-LA computation is defined as follows:

(1) $$MG\text{-}LA\left(Q,K,V\right)=\sum_{s=1}^{S}G_{s}⨀\;\left(\emptyset \left(Q_{s}\right)\left({\emptyset \left(K_{s}\right)}^{T}V_{s}\right)\right),$$

where S denotes the number of multiscale levels; $Q_{s},K_{s},V_{s}$ represent the query, key, and value for each scale, respectively; $\emptyset \left(\cdot \right)$ denotes a linearization function that transforms the conventional dot-product attention into a linear computation; $G_{s}$ denotes a learnable gating vector that emphasizes important temporal segments; and ⊙ denotes the element-wise multiplication operation.

The output of MG-LA is refined through a linear projection, followed by a residual connection to the input sequence and layer normalization for stable training. Subsequently, a feed-forward neural network (FFN) is applied to effectively learn complex patterns that are difficult to capture using a simple linear model. This enables the coarse-grained branch (CGB) to generate ${F_{i}}^{cg}$, which reflects long-term temporal dependencies, as follows:

(2) $$F_{i}^{cg}=FFN\left(LN\left(F_{i}^{MG\text{-}LA}+MaxPool\left(F_{i}\right)\right)\right),$$

where LN(·) denotes layer normalization.

FGB comprises a 1D CNN-based fine-grained temporal learning module that captures short-term and fine-grained temporal variations in the input EEG time series. First, a low-rate dropout is applied to prevent information loss and overfitting, followed by a CNN with a kernel size of 3 to learn fine patterns within short temporal windows. Batch normalization and ELU activation are then applied to enhance training stability and sensitivity to small amplitude changes. Finally, stride-1 max pooling is performed to compress the key peak signals, producing the refined short-term feature representation ${F_{i}}^{fg}$, as follows:

(3) $$F_{i}^{fg}=MaxPool\left(ELU\left(BatchNorm\left(FConv\left(DP\left(F_{i}\right)\right)\right)\right)\right),$$

where DP(·) denotes the dropout operation, and FConv(·) represents fine convolution.

MGB comprises a medium-grained temporal learning module designed to capture intermediate-range patterns between short- and long-term dynamics in an EEG time series. A relatively high dropout rate is applied to prevent overfitting, and 1D CNN filters with a kernel size of seven are used to learn medium-length temporal patterns and local features. Subsequently, average pooling is applied to compress the core information, effectively capturing high-frequency components, gradual changes, and mid-range frequency patterns. This process generates a medium-scale feature representation, ${F_{i}}^{mg}$, which contributes to the integration of multiscale information in the subsequent layers:

(4) $$F_{i}^{mg}=AvgPool\left(ELU\left(BatchNorm\left(MConv\left(DP\left(F_{i}\right)\right)\right)\right)\right),$$

where MConv(·) represents medium convolution.

In each HCT layer, the coarse-grained feature ${F_{i}}^{cg}$, medium-grained feature ${F_{i}}^{mg}$, and fine-grained feature ${F_{i}}^{fg}$ are integrated and passed as inputs to the next layer. The repeated application of this hierarchical structure leads to the accumulation of information across multiple temporal scales throughout the EEG time series, providing a foundation for learning both the global context and fine-grained temporal patterns simultaneously. The final output of the HCT is generated through a hierarchical integration process as follows:

(5) $$F_{i+1}=F_{integ}=F_{i}^{cg}+F_{i}^{mg}+F_{i}^{fg},$$

${F_{i}}^{mg}$ and ${F_{i}}^{fg}$ are subsequently used in the information refinement module, ADIP.

HCMFT introduces the previously missing medium temporal-scale learning to the conventional HCFT, enabling comprehensive learning of long-, medium-, and short-term patterns in EEG signals. This enables the precise capture of both gradual emotional transitions and instantaneous responses, enhancing the accuracy and expressiveness of emotion recognition.

#### 3.2.3. Attentive Dense Information Purification (ADIP)

In this study, we propose an ADIP module integrated with an HCMFT to effectively capture the fine-grained and hierarchical temporal dynamics inherent in EEG signals. Given the noisy characteristics of EEG data, high-resolution feature maps extracted at the MGB and FGB stages often contain a mixture of essential emotional information and irrelevant artifacts.

In a conventional DIP framework, a log-power transformation is applied by squaring the signal outputs at each time step, averaging them along the temporal dimension, and subsequently applying a logarithmic function. This operation yields a stable representation of the signal amplitude, while implicitly reflecting its frequency-domain characteristics. Nevertheless, the DIP framework lacks the capability to dynamically evaluate the temporal and channel-wise significance of features and does not selectively amplify core emotional signals in accordance with their contextual relevance.

Therefore, the ADIP module receives the feature map $F^{power}\in \;R^{C\times T}$, obtained through log-power transformation, as input and dynamically evaluates the importance of each channel and temporal segment using a self-attention-based computation. Specifically, for $F^{power}$, the query *Q*, key *K*, and value *V* matrices are computed through linear transformations, and the scaled dot-product attention mechanism is applied to derive the attention weight *A*:

(6) $$A=Softmax\left(\frac{QK^{T}}{\sqrt{d_{k}}}\right),F^{attn}=AV,$$

using $Q=F^{power}W_{Q}$, $K=F^{power}W_{K}$, and $V=F^{power}W_{V}$.

The generated attention map *A* serves as a weighted filter that highlights essential information while suppressing noise. Certain parts of the original features are preserved through a gated residual structure [37], ensuring that only the refined information is propagated to higher layers:

(7) $$F^{refined}=\sigma \left(G\right)\;⨀\;F^{attn}+\left(1-\sigma \left(G\right)\right)\;⨀\;F^{power},$$

The refined medium and fine-grained features are combined into a unified representation $F_{fusion}$ through a learnable sum fusion:

(8) $$F_{fusion}=F_{medium}^{refined}+F_{fine}^{refined},$$

The final integrated representation is passed to the subsequent Transformer encoder and MLP classifier to infer the emotional state. The integration of log-power transformation, self-attention, and gated residual mechanisms selectively amplifies key emotional signals within the EEG data while attenuating noise, thereby enhancing the emotion recognition performance relative to the conventional DIP approach.

#### 3.2.4. Feature Combination, Transformer Encoder, and MLP Classifier with Contrastive Learning

The final stage of this study involved feature combinations, where the coarse-, medium-, and fine-scale features extracted from HCMFT were integrated with the refined medium- and fine-scale features from the ADIP module into a single unified representation. This representation was then mapped to emotion labels using a Transformer encoder and an MLP classifier.

The preprocessed EEG signal $X\;\in R^{C\times T}$ undergoes data augmentation, including noise addition, temporal shifting, and filtering, to generate positive and negative pairs. A positive pair $\left(X_{i},{\;X}_{i}^{+}\right)$ comprises samples from the same subject and emotion, whereas a negative pair $\left(X_{j},{\;X}_{j}^{-}\right)$ comprises samples from a different emotion or subject.

The augmented samples are then fed into the EED. A depthwise separable convolution-based feature encoder first extracts spatiotemporal features, which are then processed by the HCMFT to hierarchically capture long-, medium-, and short-term temporal patterns. To further refine these features, the ADIP module dynamically evaluates the importance of each channel and temporal segment using self-attention and applies a gated residual mechanism to emphasize key information while suppressing noise.

The coarse branch feature, $F^{cg}$, represents long-term temporal patterns, whereas the medium and fine branch features, refined by ADIP $F_{fusion}$, contain essential medium- and short-term information. The final integrated representation is defined as follows:

(9) $$F_{concat}=Concat\left(F_{integ},F_{fusion}\right),$$

During contrastive learning-based pretraining, the integrated representation $F_{concat}$ is optimized using the InfoNCE loss to move positive pairs closer while distancing negative pairs:

(10) $$\Upsilon_{contrastive}=-log\frac{exp\left(sim\left(z_{i},z_{i}^{+}\right)/\tau \right)}{exp\left(sim\left(z_{i},z_{i}^{+}\right)/\tau \right)+\sum_{j=1}^{K}exp\left(sim\left(z_{j},z_{j}^{-}\right)/\tau \right)}\;,$$

where $z_{i}=F_{concat}$, $sim\left(a,b\right)=\frac{a\cdot b}{\left\|a\right\|⟦b⟧}$ denotes cosine similarity, τ denotes the temperature hyperparameter, and K denotes the number of negative pairs. This pretraining ensures the extraction of robust, subject-invariant EEG embeddings, even with limited labeled data.

The pretrained embedding $F_{concat}$ is subsequently fed into a Transformer encoder, producing a high-level representation *H* that captures all scales and refined information:

(11) $$H=Transformer\left(F_{concat}\right),$$

This representation *H* is input to the MLP classifier for final emotion label prediction:

(12) $$\hat{y}=MLP\left(H\right),$$

The MLP classifier comprises fully connected layers, activation functions, dropouts, and a softmax or sigmoid output layer to produce the final emotion classification.

## 4. Experiments and Results

### 4.1. Experimental Setup

In this study, we evaluated the performance of the proposed EED and CL-based EED-CL models using eight EEG datasets. To assess the generalization in cognitive tasks, we used Datasets 1 (cognitive attention), 2 (driving fatigue), and 3 (mental workload), which were previously applied in EEG-Deformer studies.

- Dataset 1 (cognitive attention) [38]: EEG signals from 26 participants performing the discrimination/selection response task were recorded using 28 channels at 1000 Hz and labeled as “attentive” or “inattentive”. Signals were bandpass filtered (0.5–50 Hz), and ocular artifacts were removed via ICA and downsampled to 200 Hz.
- Dataset 2 (driving fatigue) [39]: 32-channel EEG data (500 Hz) from 27 participants during a 90 min VR driving task were labeled as “low” or “high” fatigue. Officially preprocessed signals at 1–50 Hz were used.
- Dataset 3 (mental workload) [40]: EEG from 36 participants performing serial subtraction was recorded with 19 channels, artifacts removed with ICA, and segmented using 4 s sliding windows with 2 s overlap.

Five additional datasets were used for the real-world emotion recognition: DEAP, SEED, MAHNOB-HCI, DEBHA, and MITY.

- DEAP [41]: 32-channel EEG (512 Hz) from 32 participants watching 40 music videos was used; arousal, valence, dominance, and preference were rated from 1 to 9.
- SEED [42]: A 62-channel EEG from 15 participants watching 15 Chinese film clips (approximately 4 min) labeled as positive, neutral, or negative was used.
- MAHNOB-HCI [43]: This is a multimodal emotion dataset; only a 32-channel EEG (512 Hz) was used, with labels based on arousal and valence.
- DEBHA [44]: 36-channel EEG (1 kHz) from 30 participants watching videos inducing four emotions (happy, sad, angry, and relaxed), corresponding to the HVHA, LVHA, LVLA, and HVLA quadrants, was used.
- MITY [44]: 14-channel EEG from 16 participants watching 50 movie trailers (60 s each and five genres) was used; arousal, valence, dominance, and preference were rated from 1 to 9.

All EEG signals were bandpass filtered (0.5–50 Hz), z-score normalized, and segmented into 4 s epochs. The data augmentation included Gaussian noise injection (σ = 0.1–0.2), window shifting (window length = 1–4 s with 30~50% overlap), and time warping (±10% time distortion). These parameter ranges are consistent with commonly adopted practices in EEG augmentation studies and are suitable for preserving the essential temporal–spatial characteristics of the signals while enhancing model robustness.

The comparison models included EEGNet, EEG-Conformer, EEG-Deformer, EED, and EED-CL. EEGNet is a lightweight convolutional model with depthwise separable convolutions. The EEG-Conformer combines convolution and transformer modules to integrate local and global features, and the EEG-Deformer employs hierarchical transformers for multiscale temporal learning and serves as the baseline.

All experiments used identical training settings: AdamW optimizer, learning rate of 1 × 10^−4^, batch size of 64, weight decay of 0.01, and 1000 epochs. Cross-entropy loss with class-balanced weighting was applied to mitigate class imbalance. The models were trained on an NVIDIA RTX 4090 GPU, and their performance was evaluated using 5-Fold cross-validation, reporting the mean accuracy (%) and F1-score (%).

### 4.2. Performance on Cognitive Datasets

To evaluate whether the structural improvements of the proposed EED provide tangible performance gains over existing methods, comparative experiments were conducted with representative EEG-based models, including EEGNet, EEG-Conformer, and EEG-Deformer. Table 1 summarizes the classification performances of these models across the three cognitive EEG datasets (Attention, Fatigue, and Mental Workload). A paired t-test was conducted to compare the proposed method with other neural network models, with *p*-values below 0.05 indicating statistically significant differences.

The results indicate that the proposed EED achieved an average improvement of 3.1 percentage points in accuracy and 3.6 percentage points in F1-score compared to the original EEG-Deformer. The most substantial performance gain was observed in the Fatigue dataset, where accuracy and F1-score increased by 4.4 and 3.5 percentage points, respectively. This performance improvement is likely related to the relatively stable and consistent EEG patterns exhibited in the Fatigue state—such as increases in the θ and α frequency bands—compared to Attention or Workload, as well as the limited inter-individual variability. These characteristics are expected to facilitate the learning process of the classifier. Moreover, fatigue-related signals tend to accumulate gradually over time, which likely aligns well with the HCMFT architecture that hierarchically analyzes temporal patterns at coarse, medium, and fine scales. Their low-frequency–dominant nature may also enhance the effectiveness of the ADIP-based signal refinement. Taken together, these factors appear to explain why the Fatigue task exhibited greater performance improvements than the other tasks. Importantly, these gains are not simply due to an increase in model size; rather, they result from the hierarchical architectural design, improvements in the ADIP refinement module, and the integrated temporal–spatial pattern learning strategy.

### 4.3. Performance on Cognitive Emotion Recognition Datasets

Five EEG-based emotion datasets (DEAP, SEED, MAHNOB-HCI, DEBHA, and MITY) were used to evaluate the emotion recognition performance of the proposed EED. These datasets vary in terms of the number of participants, channel configurations, and experimental conditions, covering both controlled and naturalistic emotional responses, rendering them suitable for assessing model generalizations. The comparison models included EEGNet, EEG-Conformer, EEG-Deformer, EED, and EED-CL, which were designed to learn robust EEG feature representations under limited-label conditions.

Table 2 summarizes the accuracy and F1-score of each model across the five emotion datasets.

The proposed EED model consistently outperformed the conventional EEG-Deformer across all datasets. In SEED, where emotional states were well-defined and participant responses were highly consistent, the model achieved a 1.7 percentage-point improvement in accuracy. In MAHNOB-HCI, which includes diverse emotional stimuli, a 2.3 percentage-point enhancement was observed. Moreover, in DEAP, where inter-subject EEG variability is substantial, the model recorded a 2.1 percentage-point improvement, demonstrating its effectiveness in handling complex emotional patterns and high individual differences. Improvements of 3.7 and 4.3 percentage points were also observed in the label-scarce DEBHA and MITY datasets, respectively. This led to an average enhancement of approximately 2.8 percentage points across all five emotion datasets.

The particularly high accuracy and F1-scores observed in DEBHA and MITY can be attributed to the use of videos with clearly defined emotional content, which elicited distinct emotional responses in participants and generated pronounced variations in EEG signals, thereby enabling the model to learn distinctions between emotional states more precisely. Furthermore, the EED-CL model demonstrated substantial performance gains over EED, especially in DEBHA and MITY. This indicates that contrastive learning-based pretraining can effectively learn feature-invariant representations that are robust to inter-subject variability and noise, even in label-limited settings, thereby significantly enhancing the model’s generalization performance. Overall, the EED and EED-CL models maintained stable and high performance across diverse EEG conditions (channel count, number of subjects, stimulus type, and emotional difficulty) and provided practical accuracy in environments characterized by high inter-subject EEG variability and limited labeling, thereby demonstrating high versatility and applicability.

Conventional models tend to be biased toward majority-class predictions, and as a result, their accuracy is generally higher than their F1-score. In contrast, the proposed EED effectively enhances inter-class discriminability through multi-scale spatiotemporal feature modeling, while EED-CL further introduces contrastive learning to explicitly encourage balanced and discriminative representations among samples. This learning strategy improves class-wise precision and recall simultaneously, thereby promoting a more balanced class-wise prediction distribution. Consequently, the harmonic mean of precision and recall (F1-score) tends to surpass the overall accuracy, indicating improved robustness in emotion recognition performance.

### 4.4. Ablation Study

An ablation study was conducted to quantitatively evaluate the contribution of each architectural component of the proposed EED under the contrastive learning (CL) framework, referred to as EED-CL, to the overall model performance. The experiments were performed on the DEAP dataset while maintaining identical training configurations across all models, including the learning rate, batch size, optimizer, and loss function. Starting from the EED-CL baseline, key modules were selectively removed or replaced to construct variant models, enabling a systematic analysis of the contribution of each component while keeping the CL strategy unchanged.

Specifically, four ablation variants were designed as follows:

- w/o DSC-SFE: The DSC-SFE module in EED-CL was replaced with the standard SFE to analyze the contribution of the depthwise structure in improving parameter efficiency and decoupling spatial–temporal interactions across EEG channels.
- w/o HCMFT: The proposed HCMFT module was replaced with the HCFT to assess the effectiveness of multiscale temporal integration that jointly learns long-term, mid-term, and fine-grained EEG patterns under the CL setting.
- w/o ADIP: The ADIP module was removed, applying only the DIP mechanism without dynamic refinement of intermediate feature maps. This configuration was used to evaluate the role of ADIP in noise suppression, salient feature enhancement, and improved classification performance within the EED-CL framework.
- w/o Transformer encoder (TE): The feature maps extracted from HCMFT and ADIP were combined and directly fed into an MLP classifier, without passing through the Transformer encoder, to examine the contribution of transformer-based spatiotemporal feature integration to the overall performance improvement in EED-CL.

Through these four ablation experiments, the complementary roles of each component in the EED-CL were quantitatively assessed. The results validate that the multiscale temporal integration of HCMFT, feature refinement capability of ADIP, and the spatial–temporal disentanglement mechanism of the depthwise encoder play crucial roles in enhancing emotion recognition accuracy, even when contrastive learning is employed.

Table 3 summarizes the results of the ablation study conducted on the DEAP dataset, providing a quantitative evaluation of how the removal or replacement of each component in EED-CL affects overall EEG-based emotion recognition performance.

Table 3 shows that the EED-CL model, which includes all modules, achieves the highest performance, with an Accuracy of 82.5% and an F1-score of 82.7%. To assess the contribution of each component, we compared the EED-CL model with variants in which specific modules were removed or replaced. First, when the DSC-SFE module was removed and replaced with SFE, ΔAccuracy decreased by 1.6 percentage points and ΔF1-score by 1.8 percentage points. This indicates that the depthwise separable convolution (DSC) structure efficiently separates spatial and temporal interactions across channels, thereby contributing to improved emotion recognition performance while maintaining parameter efficiency. Next, replacing HCMFT with HCFT resulted in ΔAccuracy of −3.1 percentage points and ΔF1-score of −3.7 percentage points. This highlights the importance of the multiscale hierarchical structure in integrating long-term, mid-term, and fine-grained temporal patterns from EEG signals. Furthermore, removing the ADIP module led to ΔAccuracy of −2.4 percentage points and ΔF1-score of −2.4 percentage points. This demonstrates that ADIP effectively separates and enhances key emotion-related information from noise in intermediate feature maps, contributing to representation learning and classification performance. Finally, when the Transformer encoder was removed and replaced with a simple MLP, ΔAccuracy decreased by 4.2 percentage points and ΔF1-score by 5.4 percentage points. This emphasizes that Transformer-based feature integration and spatiotemporal pattern learning play the most critical role in improving model performance by effectively combining multiscale features extracted from EEG signals. In summary, the performance degradation caused by the removal of each module suggests that the contributions of the modules can be ranked in the following order: the Transformer encoder has the largest contribution, followed by HCMFT, then ADIP, and finally DSC-SFE. These results indicate that the overall architecture supports synergistic learning of spatial, temporal, and multiscale key information from EEG signals.

To further examine the effectiveness of HCMFT and the impact of different attention mechanisms, an additional experiment was conducted on the DEAP dataset, focusing on the CGB of HCMFT. Table 4 summarizes the results.

The evaluated attention mechanisms included conventional MSA, randomized feature-based linearized attention performer (FAVOR+) [45], gated linear attention (GLA) [46], and the proposed multiscale gated linear attention (MG-LA).

All models were trained under identical configurations and evaluated using leave-one-subject-out (LOSO) cross-validation. The model performance was assessed in terms of classification metrics (accuracy and F1-score), computational cost (FLOPs), and effective attention weight (EAW), which measure the attention focus on emotionally salient temporal segments. The results demonstrate that the proposed HCMFT architecture achieves an effective balance between temporal modeling efficiency and emotional feature sensitivity, validating its contribution to the superior performance of the EED. Table 4 presents the results of the experiments.

Experimental results show that the proposed MG-LA exhibits the most balanced characteristics in terms of performance, computational efficiency, and effective attention width (EAW) for DEAP-based EEG emotion recognition. MG-LA achieved a Macro F1 of 82.7% and an AUC of 85.4%, slightly lower than those of MSA, while its GFLOPs per sample was 0.865, substantially lower than MSA (2.20) and comparable to GLA (0.855). This improvement in computational efficiency can be attributed to MG-LA’s design, which combines a linearized attention mechanism with multiscale gating, thereby allowing computational cost to scale linearly with sequence length. MG-LA also achieved the highest EAW (70 ms), reflecting its ability to focus on emotion-relevant EEG segments through the gating and multiscale design. In comparison, MSA, which uniformly attends to all tokens via a quadratic softmax structure, exhibited higher computational cost and a relatively dispersed EAW of 65 ms, potentially diluting critical segment information. Performer, although efficient due to linear attention, showed limited representational capacity from softmax approximation, resulting in a lower EAW of 48 ms. GLA, incorporating gating and linearization, achieved an EAW of 63 ms, improving critical segment capture compared to Performer. Overall, MG-LA, through its multiscale and gating structure, simultaneously optimizes performance, computational efficiency, and attention focus, thereby demonstrating both real-time applicability and interpretability for DEAP-based EEG emotion recognition.

Table 5 presents a comparison of the model parameter count, computational cost (FLOPs), and performance between the conventional SFE (shallow feature encoder) and the proposed DSC-SFE (depthwise separable convolution-based SFE). The results show that DSC-SFE reduces the number of parameters by over 20-fold and decreases FLOPs by over 36-fold compared to the original SFE. Despite this substantial model simplification, the DSC-SFE-based EED-CL achieves an accuracy improvement from 77.5% to 79.6% and a F1-score increase from 76.8% to 78.9%. This improvement can be attributed to the use of depthwise separable convolution, which effectively reduces redundant computations across EEG channels while preserving discriminative spatial and channel-wise feature representations. Overall, these results demonstrate that the introduction of DSC-SFE enables effective model lightweighting and computational efficiency while simultaneously improving emotion recognition performance on the DEAP dataset, highlighting its suitability for efficient EEG-based emotion recognition systems.

### 4.5. Effect of Contrastive Learning

To quantitatively evaluate the impact of CL-based pretraining on EEG emotion recognition under limited-label conditions, we systematically reduced the label ratio to 100%, 50%, and 25%, comparing the performances of EED and EED-CL. Experiments were conducted on the DEBHA dataset, maintaining all training settings—including the learning rate, batch size, optimizer, and loss function—identical to ensure a fair comparison. Table 6 summarizes the accuracy and F1-score for both models across different label ratios on the DEBHA dataset.

Even under full-label (100%) conditions, EED-CL demonstrated slightly improved performance compared to EED. As the label ratio decreased, the performance gap between the two models became more pronounced. Notably, when only 25% of the labels were available, EED-CL outperformed EED by 10.5 percentage points in accuracy and 11.8 percentage points in F1-score, clearly demonstrating that CL-based pretraining effectively enhances the stability and generalization of EEG representations in data-constrained scenarios.

When the label ratio was reduced from 100% to 25%, the EED-only model exhibited decreases of 13.1 percentage points in Accuracy and 12.9 percentage points in F1-score, whereas the EED-CL model only decreased by 6.3 percentage points and 5.8 percentage points, respectively, indicating that performance degradation was relatively smaller under limited label conditions. This is because, when labels are scarce, individual variability and noise in EEG signals have a greater impact on learning. The EED model relies solely on label information to structure the feature space, so class boundaries become ambiguous in low-label scenarios, leading to overfitting and sharp performance drops. In contrast, EED-CL leverages contrastive learning-based pretraining to learn stable and generalizable EEG representations without labels and generates noise-robust embeddings through augmentation, thereby maintaining class structure even under limited label conditions. Consequently, EED-CL exhibits relatively smaller performance degradation in label-scarce settings and offers advantages in cross-subject generalization and feature space stability.

In summary, by integrating EED’s structural strengths—multiscale temporal pattern learning via HCMFT and feature refinement via ADIP—with contrastive learning-based pretraining, the model simultaneously improves spatiotemporal feature learning and inter-class separation, leading to significantly enhanced emotion recognition performance. These results empirically demonstrate that contrastive learning enhances generalization and robustness in EEG-based emotion recognition under limited-label conditions, providing practical evidence for addressing label scarcity in real-world applications.

## 5. Discussion and Limitations

In this study, we proposed the EED and its combination with CL-based pretraining, termed EED-CL, to enhance EEG-based emotion recognition. Several key findings were observed during the experiments.

First, the structural enhancements of the EED, encompassing the DSC-SFE, HCMFT, and ADIP modules, effectively captured both spatial and temporal EEG patterns while mitigating noise, thereby yielding a clear performance improvement relative to the baseline EEG-Deformer. Ablation studies demonstrated that removing HCMFT caused the largest performance drop, confirming the importance of multiscale temporal integration. Excluding ADIP reduced the intermediate feature refinement and lowered the F1-scores, whereas removing the depthwise encoder degraded both the computational efficiency and fine-grained pattern recognition. These results indicate that each module contributes independently and complementarily to the overall performance.

Second, CL-based pretraining proved to be particularly effective under limited-label conditions. For datasets with scarce labels, such as DEBHA and MITY, EED-CL significantly outperformed EED alone in terms of both accuracy and F1-score. Experiments with label ratios of 100%, 50%, and 25% demonstrated that EED-CL mitigated performance degradation by learning robust feature representations and effectively handling intersubject variability and noise. This indicates that CL enables stable EEG representation learning and preserves generalization even with minimal labeled data.

Third, the combination of structural advantages of EED and CL pretraining enhanced not only spatiotemporal feature learning but also interclass representation separation, facilitating clear discrimination of diverse emotional states. The integration of multiscale HCMFT and ADIP emphasized critical channels and fine-grained temporal patterns, partially improving model interpretability.

However, this study has several limitations. Most datasets were collected from healthy adults in controlled environments and require further validation for different age groups, neurological conditions, and naturalistic settings. The complexity of the EED and EED-CL may require optimization for real-time applications in low-resource settings. Furthermore, as datasets mainly rely on discrete emotion labels, their performance in continuous or multidimensional emotional spaces (e.g., valence and arousal) remains unverified. The effectiveness of CL can vary depending on the dataset size, class imbalance, and intersubject variability, whereas the neurophysiological validity of the intermediate features emphasized by ADIP warrants further investigation. Furthermore, comparisons with recent self-supervised methods should be conducted to fully evaluate the relative advantages of the proposed approach.

The EED-CL model is relatively complex due to its hierarchical architecture and integrated modules. Preliminary analyses using the SEED dataset (62 channels, 200 Hz, segmented into 256 samples per window) were conducted to evaluate the model’s execution time and memory usage under standard GPU conditions. The results indicated that the hierarchical transformer structure (HCMFT) and ADIP module contributed most to the computational cost, whereas the depthwise separable convolution remained relatively efficient. Forward passes with a batch size of 32 required approximately 20–33 ms, with total memory usage around 220 MB. These findings suggest that the model is suitable for offline research applications; however, further optimization would be necessary for deployment in real-time or resource-constrained environments.

Future work will include evaluating and optimizing the model across diverse EEG channel configurations and sensor placements, exploring real-time and online learning scenarios for applications in HCI and well-being monitoring, and extending the approach to multimodal biosignals (e.g., ECG, GSR, and facial expressions) to further improve the recognition accuracy and robustness. Moreover, optimizing CL-based pretraining for various label-scarce scenarios can minimize annotation costs while maintaining high performance in practical environments.

In summary, our study demonstrated that EED combined with CL-based pretraining effectively enhances spatiotemporal pattern learning, noise suppression, intersubject variability handling, and generalization under limited-label conditions for EEG emotion recognition. Simultaneously, areas of improvement were identified, including dataset diversity, real-time applicability, emotion expression coverage, dependency on CL, and model interpretability.

## 6. Conclusions

In this study, we proposed an EED-CL model, which integrates EED with CL to enhance EEG-based emotion recognition. EED utilizes the DSC-SFE, HCMFT, and ADIP modules to effectively capture spatiotemporal EEG patterns and suppress noise, thereby providing more stable and reliable emotion classification performance compared to conventional EEG-Deformer approaches. In particular, CL-based pretraining enables the model to learn robust and generalizable feature representations even with limited labeled data, maintaining consistent performance across diverse annotation conditions.

Ablation studies confirmed that HCMFT plays a critical role in integrating multiscale temporal information; ADIP enhances discriminative capability through intermediate feature refinement; and the depthwise encoder simultaneously improves spatiotemporal pattern learning and computational efficiency. These results demonstrate that the proposed architecture achieves meaningful performance gains not merely by increasing model size or complexity, but through a design informed by physiological characteristics and temporal structures.

Moreover, EED-CL combines stability, interpretability, and scalability, making it suitable for construction of reliable EEG-based emotion recognition systems even in data-constrained environments. These characteristics suggest its potential applicability not only in laboratory settings but also in practical applications such as human–computer interactions, emotion-aware recommendation systems, and mental health monitoring.

## Figures and Tables

**Table 1:** Comparison of classification performance on cognitive datasets (The best results are marked in bold).

Model	Attention (ACC/F1)	Fatigue (ACC/F1)	Workload (ACC/F1)
EEGNet [21]	73.9 ± 10.2/ 72.5 ± 10.5 (0.021)	72.3 ± 10.0/ 70.5 ± 10.6 (0.017)	65.1 ± 10.6/ 61.5 ± 10.8 (0.023)
EEG-Conformer [14]	78.1 ± 11.1/ 78.6 ± 10.7 (0.017)	74.4 ± 10.2/ 72.5 ± 10.7 (0.015)	68.6 ± 11.2/ 65.5 ± 10.6 (0.018)
EEG-Deformer [16]	81.4 ± 9.8/ 80.8 ± 10.3 (0.012)	77.9 ± 10.1/ 76.0 ± 9.9 (0.011)	72.2 ± 9.7/ 69.2 ± 10.2 (0.009)
**EED (ours)**	**84.1** ± **9.5/** **83.8** ± **9.9** **(-)**	**82.3** ± **9.2/** **79.5** ± **10.1** **(-)**	**74.5** ± **9.9/** **73.5** ± **9.5** **(-)**

Values in parentheses represent the *p*-value of the accuracy.

**Table 2:** Comparison of EEG-Based Emotion Recognition Models (Accuracy/F1-score).

Model	DEAP (High)	SEED (Low)	MAHNOB-HCI (High)	DEBHA (Medium)	MITY (Medium)
EEGNet	70.8 ± 11.3/ 70.1 ± 11.0 (0.038)	77.6 ± 11.2/ 78.1 ± 10.8 (0.037)	72.4 ± 11.7/ 71.9 ± 11.1 (0.032)	78.1 ± 11.2/ 77.5 ± 10.7 (0.029)	75.3 ± 10.6/ 74.8 ± 10.4 (0.028)
EEG-Conformer	74.4 ± 9.8/ 74.2 ± 10.1 (0.026)	81.3 ± 12.1/ 81.7 ± 11.2 (0.024)	74.1 ± 10.2/ 73.6 ± 10.3 (0.022)	81.4 ± 11.2/ 81.8 ± 10.7 (0.017)	78.8 ± 9.8/ 78.4 ± 10.3 (0.019)
EEG-Deformer	77.5 ± 8.7/ 76.8 ± 9.1 (0.018)	85.4 ± 9.5/ 84.9 ± 10.2 (0.017)	78.1 ± 8.9/ 77.9 ± 9.8 (0.019)	85.8 ± 11.2/ 85.2 ± 10.8 (0.016)	81.2 ± 8.7/ 81.6 ± 9.2 (0.015)
EED	79.6 ± 8.2/ 78.9 ± 7.8 (0.016)	87.1 ± 8.6/ 87.3 ± 9.2 (0.012)	80.4 ± 10.1/ 80.8 ± 9.5 (0.014)	89.5 ± 9.6/ 88.7 ± 9.1 (0.013)	85.5 ± 8.5/ 84.9 ± 8.7 (0.015)
EED-CL	82.5 ± 8.5/ 82.7 ± 7.8 (-)	89.6 ± 9.2/ 89.7 ± 8.4 (-)	83.5 ± 10.2/ 84.3 ± 9.6 (-)	93.2 ± 8.6/ 93.4 ± 9.2 (-)	90.1 ± 10.1/ 90.5 ± 8.9 (-)

Values in parentheses represent the *p*-value of the accuracy.

**Table 3:** Results of the Extended EEG-Deformer (EED) Ablation Study Based on the DEAP Dataset.

Ablation Model	Accuracy	ΔAccuracy	F1-Score	ΔF1-Score
EED-CL	82.5	0.0	82.7	0.0
w/o HCMFT	79.4	−3.1	79.2	−3.7
w/o ADIP	80.1	−2.4	80.5	−2.4
w/o DSC-SFE	80.9	−1.6	81.1	−1.8
w/o TE	78.3	−4.2	77.5	−5.4

**Table 4:** Comparison of Attention Mechanisms on the DEAP Dataset (LOSO Mean ± Standard Deviation).

HCMFT Variant	Macro F1	AUC	GFLOPs/Sample	EAW (ms)
MSA	83.5 ± 1.8	85.3 ± 1.4	2.20	65
Performer	77.6 ± 1.6	80.5 ± 1.3	0.40	48
GLA	81.3 ± 1.5	84.2 ± 1.2	0.855	63
**MG-LA (proposed)**	**82.7** ± **1.4**	**85.4** ± **1.1**	**0.865**	**70**

**Table 5:** Comparison of Model Complexity and Performance between SFE and DSC-SFE on the DEAP Dataset.

Metric	EEG-Deformer	EED
Feature Encoder	SFE (2D CNN)	DSC-SFE (Depthwise Separable Conv)
Number of Parameters	263,200	12,800
FLOPs	$209\times 10^{7}$	$57.5\times 10^{6}$
Accuracy(%)	77.5	79.6
F1-score	76.8	78.9

**Table 6:** Performance Comparison of EED and EED-CL under Different Label Ratios on the DEBHA Dataset.

Label Ratio	Model	Accuracy	F1-Score
100%	EED	89.5 ± 2.0	88.7 ± 2.2
100%	EED-CL	93.2 ± 1.8	93.4 ± 2.0
50%	EED	84.2 ± 2.5	83.9 ± 2.8
50%	EED-CL	91.5 ± 2.3	91.8 ± 2.5
25%	EED	76.4 ± 3.5	75.8 ± 3.8
25%	EED-CL	86.9 ± 3.0	87.6 ± 3.2

## Data Availability

Dataset 1 (cognitive attention) is available at https://github.com/JaeyoungShin/simultaneous_EEG-NIRS (accessed on 5 November 2025). Dataset 2 (driving fatigue) is available at https://figshare.com/articles/dataset/Multi-channel_EEG_recordings_during_a_sustained-attention_driving_task_preprocessed_dataset_/7666055/3 (accessed on 5 November 2025). Dataset 3 (mental workload) is available at https://physionet.org/content/eegmat/1.0.0/ (accessed on 5 November 2025). The SEED dataset is available at https://bcmi.sjtu.edu.cn/home/seed/ (accessed on 8 August 2023). Raw data from the DEBHA and MITY datasets can be obtained by writing a formal email to Hyoung-Gook Kim.

## Abbreviations

ADIP	Attentive dense information purification
AUC	Area under the ROC curve
CGB	Coarse-grained branch
CL	Contrastive learning
CNN	Convolutional neural network
DIP	Dense information purification
EAW	Effective attention weight
EED	Extended EEG-deformer
ELU	Exponential linear unit
FFN	Feed-forward neural network
FGB	Fine-grained branch
GLA	Gated linear attention
GRUs	Gated recurrent units
GSRs	Galvanic skin responses
HCFT	Hierarchical coarse-to-fine transformer
HCMFT	Hierarchical coarse-to-medium-to-fine transformer
LOSO	Leave-one-subject-out
LSTM	Long short-term memory
MGB	Medium-grained branch
MSA	Multihead self-attention
RNN	Recurrent neural network
ACC	Accuracy
AdamW	Adaptive moment estimation with weight decay
ECGs	Electrocardiograms
EEG	Electroencephalography
EMG	Electromyography
EOG	Electrooculography
HCI	Human–computer interaction
HCT	Hierarchical coarse-to-fine transformer
HVHA	High valence high arousal
HVLA	High valence low arousal
LVHA	Low valence high arousal
LVLA	Low valence low arousal
ICA	Independent component analysis
MLP	Multi-layer perceptron
MSIT	Ministry of Science and ICT
NRF	National Research Foundation of Korea
ROC	Receiver operating characteristic
DSC	Depthwise separable convolution
SFE	Shallow feature encoder
VR	Virtual reality
